# Supplementary material for: Complete Genome Sequence of Two Deep-Sea Streptomyces Isolates from Madeira Archipelago and Evaluation of Their Biosynthetic Potential
Source: Mar Drugs. 2021 Nov 1;19(11):621. doi: 10.3390/md19110621 (PMC8622039; doi:10.3390/md19110621)
Supplement: Supplementary file 1 [file marinedrugs-19-00621-s001.zip › marinedrugs-1408858-Supplementary.pdf]

marine drugs

Supplementary material

Complete genome sequence of two deep-sea *Streptomyces* isolates from Madeira archipelago and evaluation of their biosynthetic potential

Pedro Albuquerque [1,2], Inês Ribeiro [3,4], Sofia Correia [3], Ana Paula Mucha [3,5], Paula Tamagnini [1,2,5], Andreia Braga-Henriques [6,7], Maria de Fátima Carvalho [3,4] and Marta V. Mendes [1,2*]

1 i3S - Instituto de Investigação e Inovação em Saúde, Universidade do Porto, Rua Alfredo Allen 208, 4200-135 Porto, Portugal.
2 IBMC - Instituto de Biologia Molecular e Celular, Universidade do Porto, Rua Alfredo Allen 208, 4200-135 Porto, Portugal.
3 CIIMAR - Interdisciplinary Centre of Marine and Environmental Research, University of Porto, Terminal de Cruzeiros do Porto de Leixões, Avenida General Norton de Matos s/n, 4450-208, Matosinhos, Portugal.
4 ICBAS - Instituto de Ciências Biomédicas Abel Salazar, Universidade do Porto, Rua de Jorge Viterbo Ferreira 228, 4050-313 Porto, Portugal.
5 Departamento de Biologia, Faculdade de Ciências, Universidade do Porto, Rua do Campo Alegre, Edifício FC4, 4169-007 Porto, Portugal.
6 OOM - Oceanic Observatory of Madeira & MARE - Marine and Environmental Sciences Centre, ARDITI - Agência Regional para o Desenvolvimento da Investigação Tecnologia e Inovação, Caminho da Penteada, 9020-105 Funchal, Madeira, Portugal.
7 Regional Directorate for Fisheries, Regional Secretariat for the Sea and Fisheries, Government of the Azores, Rua Cônsul Dabney - Colónia Alemã, 9900-014 Horta, Azores, Portugal.

* Correspondence: mvm@ibmc.up.pt

Table S1: Comparison of COG functional categories. Values are displayed as: hit percentage (number of hits).

	MA3_2.13	S07_1.15	*S. coelicolor* A3(2)	*S. avermitilis* MA4680	*S. griseus* NBRC 13350
**CELLULAR PROCESSES AND SIGNALING**					
**[D]** Cell cycle control, cell division, chromosome partitioning	1,2 (75)	1,4 (93)	1,2 (101)	1,2 (90)	0,9 (64)
**[M]** Cell wall/membrane/envelope biogenesis	4,3 (273)	4,3 (281)	4,3 (353)	4,3 (326)	4,5 (314)
**[N]** Cell motility	0,3 (21)	0,5 (32)	0,5 (38)	0,5 (39)	0,2 (15)
**[O]** Post-translational modification, protein turnover, and chaperones	2,4 (156)	2,4 (157)	2,5 (206)	2,6 (192)	2,7 (187)
**[T]** Signal transduction mechanisms	5,0 (323)	6,2 (402)	6,4 (524)	6,1 (457)	5,8 (404)
**[U]** Intracellular trafficking, secretion, and vesicular transport	1,2 (76)	1,2 (81)	1,1 (92)	1,1 (83)	0,9 (60)
**[V]** Defense mechanisms	2,0 (130)	2,1 (139)	2,2 (180)	2 (154)	2,4 (166)
**[W]** Extracellular structures	0,0 (1)	0,0 (1)	0,0 (1)	0,0 (2)	0,0 (0)
**[Y]** Nuclear structure	0,0 (0)	0,0 (0)	0,0 (0)	0,0 (0)	0,0 (0)
**[Z]** Cytoskeleton	0,1 (6)	0,1 (4)	0,1 (9)	0,1 (4)	0,1 (4)
**INFORMATION STORAGE AND PROCESSING**					
**[A]** RNA processing and modification	0,0 (3)	0,1 (5)	0,0 (4)	0,0 (3)	0,0 (1)
**[B]** Chromatin structure and dynamics	0,1 (4)	0,0 (3)	0,0 (4)	0,0 (3)	0,0 (3)
**[J]** Translation, ribosomal structure and biogenesis	3,4 (219)	3,3 (215)	3,1 (256)	3,2 (237)	3,2 (223)
**[K]** Transcription	11,9 (765)	10,0 (646)	12,5 (1016)	11,6 (870)	11,1 (771)
**[L]** Replication, recombination and repair	4,8 (307)	4,3 (278)	4,6 (378)	4,5 (337)	3,9 (275)
**METABOLISM**					
**[C]** Energy production and conversion	4,9 (312)	5,1 (332)	5,1 (414)	5,0 (372)	4,8 (336)
**[E]** Amino acid transport and metabolism	8,5 (544)	7,5 (487)	7,9 (647)	9,0 (674)	8,9 (620)
**[F]** Nucleotide transport and metabolism	1,9 (122)	1,7 (112)	1,8 (145)	1,8 (134)	1,7 (119)
**[G]** Carbohydrate transport and metabolism	8,7 (559)	6,9 (450)	8,5 (695)	8,1 (606)	6,9 (483)
**[H]** Coenzyme transport and metabolism	3,1 (197)	3,0 (194)	2,9 (239)	2,7 (200)	2,9 (200)
**[I]** Lipid transport and metabolism	4,0 (256)	4,0 (260)	4,2 (340)	4,8 (361)	4,4 (307)
**[P]** Inorganic ion transport and metabolism	5,7 (365)	4,0 (262)	4,7 (387)	4,3 (323)	5,7 (398)
**[Q]** Secondary metabolites biosynthesis, transport, and catabolism	5,2 (333)	3,4 (222)	3,8 (306)	4,8 (360)	4,3 (302)
**POORLY CHARACTERIZED**					
**[S]** Function unknown	13,6 (874)	14,5 (939)	14,7 (1202)	15,1 (1136)	19,1 (1331)
No COG in database	9,0 (579)	13,1 (853)	3,3 (271)	3,1 (236)	2,6 (182)

**Total number of proteins**	6412	6492	8154	7514	6968

Table S2: Genomic islands identified in the genomes of MA3_213 and S07_1.15

Isolate	Genomic Island	Island start	Island end	Length (bp)	Method	Relevant features
	1	4663	32547	27884	IslandPath-DIMOB	
	2	607493	616900	9407	IslandPath-DIMOB	
	3	1663087	1678018	14931	IslandPath-DIMOB	Mobile element protein
	4	2600374	2622924	22550	IslandPath-DIMOB	CRISPR-associated proteins and Mobile element proteins
	5	3009903	3023877	13974	IslandPath-DIMOB	
	6	3041928	3071784	29856	IslandPath-DIMOB	Integrase
	7	3092105	3135603	43498	IslandPath-DIMOB	Mobile element protein
	8	3226506	3232914	6408	IslandPath-DIMOB	Transposase
**MA3_2.13**	9	3273702	3290977	17275	IslandPath-DIMOB	Mobile element protein and Integrases
	10	3382536	3395029	12493	IslandPath-DIMOB	Phage-related proteins and Excisionase
	11	3809724	3826244	16520	IslandPath-DIMOB	
	12	4092154	4107121	14967	IslandPath-DIMOB	Integrase and Excisionase
	13	4703582	4751050	47468	IslandPath-DIMOB	CRISPR-associated proteins, Mobile element protein and Transposase
	14	4759298	4800157	40859	IslandPath-DIMOB	
	15	4925394	4942767	17373	IslandPath-DIMOB	Phage-related protein and Mobile element proteins
	16	6652236	6660866	8630	IslandPath-DIMOB	Mobile element protein
	17	6709831	6734986	25155	IslandPath-DIMOB	Transposases
	1	820799	825992	5193	SIGI-HMM	
	2	865249	870073	4824	SIGI-HMM	
	3	884459	890019	5560	SIGI-HMM	
	4	912766	918930	6164	SIGI-HMM	
		923285	976748	53463	IslandPath-DIMOB	CRISPR-associated proteins, Mobile element protein and Transposase
	5	928312	934464	6152	SIGI-HMM	CRISPR-associated proteins
		937240	954011	16771	SIGI-HMM	Mobile element protein and Transposase
	6	1022226	1065261	43035	IslandPath-DIMOB	Mobile element proteins and Integrase
		1053820	1060195	6375	SIGI-HMM	Integrase
	7	2067148	2071285	4137	SIGI-HMM	
		2069658	2084180	14522	IslandPath-DIMOB	Transposase
	8	2240674	2255374	14700	SIGI-HMM	
	9	2625726	2634395	8669	IslandPath-DIMOB	Integrase
	10	2648868	2652958	4090	SIGI-HMM	
	11	2790463	2808442	17979	IslandPath-DIMOB	Phage-related protein
	12	2983614	2988106	4492	SIGI-HMM	
	13	3179595	3191350	11755	IslandPath-DIMOB	
**S071_1.15**	14	3298107	3318922	20815	IslandPath-DIMOB	Mobile element proteins
	15	3912705	3918872	6167	SIGI-HMM	
	16	4021957	4041330	19373	IslandPath-DIMOB	Integrase
	17	4247704	4253460	5756	IslandPath-DIMOB	Mobile element proteins and Transposases
		4247704	4255562	7858	SIGI-HMM	Mobile element proteins and Transposases
	18	4658659	4664088	5429	SIGI-HMM	
	19	4921286	4925629	4343	SIGI-HMM	
	20	5029398	5033675	4277	SIGI-HMM	CRISPR-associated proteins
	21	5037727	5045680	7953	SIGI-HMM	CRISPR-associated protein
	22	5079814	5085097	5283	SIGI-HMM	
	23	5097089	5102713	5624	SIGI-HMM	
	24	5163503	5168283	4780	SIGI-HMM	
	25	5852832	5871205	18373	SIGI-HMM	
	26	7089390	7093434	4044	SIGI-HMM	Mobile element protein
	27	7155497	7163263	7766	SIGI-HMM	
	28	7167448	7174408	6960	SIGI-HMM	CRISPR-associated protein
		7181331	7189028	7697	SIGI-HMM	
	29	7181503	7250325	68822	IslandPath-DIMOB	Mobile element proteins
		7211301	7215383	4082	SIGI-HMM	

Table S3: Identified prophage regions

Isolate	Region	Region Length	Completeness	Score	# Total Proteins	Region Position	Mo[illegible]
**MA3 2.13**	1	18.9Kb	incomplete	30	15	3377225-3396181	PHAGE_Caulob_C[illegible]
**S071 1.15**	1	8.3Kb	incomplete	10	11	422931-431231	PHAGE_Strept_Idi[illegible]
	2	11.1Kb	incomplete	20	9	969242-980378	PHAGE_Mycoba_A[illegible]
	3	6.4Kb	incomplete	10	10	2576920-2583329	PHAGE_Strept_Da[illegible]
	4	6.6Kb	incomplete	10	10	3942773-3949468	PHAGE_Strept_Idi[illegible]
	5	9.6Kb	incomplete	10	9	5050356-5060016	PHAGE_Gordon_G[illegible]
	6	6.2Kb	incomplete	30	8	5257587-5263873	PHAGE_Strept_Pic[illegible]
	7	8.6Kb	incomplete	10	9	6644103-6652740	PHAGE_Strept_Pic[illegible]
	8	6.4Kb	incomplete	20	7	7055804-7062223	PHAGE_Synech_S_[illegible]

Table S4: Average nucleotide identity (ANI; %) values between isolates MA3_2.13, S07_1.15 and their closest strains as determined by the WGS-based tree.

	1	2	3	4	5	6	7	8	9	10	11	12	13	14	15	16	17	18	19	20	21	22	23	24	25	26	27	28	29	30	31
1) *Streptomyces autolyticus* CGMCC0516		81,22	76,39	76,32	75,41	98,49	82,91	75,24	97,88	83,24	97,90	76,49	78,54	78,40	83,08	77,81	77,03	77,75	98,41	90,21	76,74	76,39	90,70	90,71	76,47	78,48	90,26	76,24	76,86	78,91	78,25
2) *Streptomyces bingchenggensis* BCW-1	81,67		76,58	76,75	75,13	81,70	81,25	75,72	81,68	81,59	81,67	76,40	78,36	78,33	81,47	78,27	76,89	78,30	81,68	81,88	76,55	76,54	81,88	81,86	76,60	78,27	81,74	76,44	76,66	78,60	78,48
3) *Streptomyces* sp CMB-StM0423	77,24	77,16		77,03	75,93	77,15	77,22	76,50	77,12	77,39	77,12	76,57	77,66	77,66	77,36	77,53	76,81	77,44	77,14	77,18	76,53	94,43	77,13	77,12	94,79	77,46	77,10	77,15	76,71	77,65	77,59
4) *Streptomyces albus* CAS922	77,46	77,61	76,99		76,42	77,44	77,60	76,66	77,46	77,74	77,47	77,25	77,87	78,21	77,59	77,70	78,24	77,58	77,37	77,48	77,21	76,95	77,28	77,40	77,05	77,81	77,49	80,69	77,45	78,16	77,70
5) MA3_2.13	75,90	75,36	75,46	75,54		75,86	75,47	**77,90**	75,52	75,64	75,52	77,35	75,55	76,49	75,57	75,42	75,88	75,39	75,83	75,47	77,41	75,48	75,44	75,44	75,50	75,51	75,42	75,57	77,51	76,71	75,34
6) *Streptomyces solisilvae* HNM0141	97,84	81,00	76,17	76,27	75,24		82,55	75,28	98,07	82,86	98,08	76,29	78,06	78,15	82,76	77,64	76,89	77,56	98,33	89,66	76,44	76,20	90,28	90,29	76,29	78,04	89,83	76,24	76,45	78,65	78,04
7) *Streptomyces* sp NA02950	83,87	81,81	76,82	77,29	75,91	83,81		76,32	83,79	83,53	83,78	76,94	78,98	78,53	83,32	78,81	76,98	78,68	83,76	83,86	76,99	76,84	83,92	83,98	77,02	78,89	83,88	76,97	77,16	78,69	78,96
8) *Streptomyces* sp. SCSIO 3032	76,33	76,39	76,36	76,47	78,34	76,32	76,44		76,34	76,74	76,34	77,99	76,79	76,85	76,62	76,67	76,50	76,57	76,33	76,30	78,04	76,36	76,25	76,23	76,38	76,74	76,30	76,54	78,24	77,12	76,48
9) *Streptomyces malaysiensis* DSM 4137	97,68	81,18	76,23	76,39	75,09	98,47	82,81	75,36		83,32	99,98	76,37	78,35	78,26	83,09	77,76	76,84	77,64	98,37	90,06	76,53	76,25	90,69	90,61	76,31	78,24	90,20	76,34	76,67	78,67	78,20
10) *Streptomyces* sp Rer75	83,43	81,40	76,56	76,74	75,39	83,37	82,76	75,86	83,46		83,46	76,68	78,33	78,63	94,70	78,34	77,18	78,18	83,35	83,60	76,77	76,62	83,72	83,73	76,70	78,34	83,63	76,52	76,78	79,05	78,72
11) *Streptomyces malaysiensis* DSM 4137	97,64	81,21	76,24	76,36	75,02	98,48	82,84	75,45	99,97	83,31		76,40	78,42	78,26	83,12	77,88	76,89	77,64	98,37	90,09	76,51	76,30	90,72	90,66	76,29	78,31	90,20	76,41	76,63	78,69	78,14
12) *Streptomyces xiamenensis* MCCC:1A01550	77,17	77,09	76,70	77,29	77,70	77,14	77,28	78,09	77,15	77,27	77,15		77,41	77,41	77,20	77,34	76,64	77,23	77,17	77,09	97,87	76,81	77,09	77,01	76,79	77,31	77,06	77,26	86,99	77,35	77,14
13) *Streptomyces buecherae* AC541	79,29	79,05	77,47	77,61	76,16	79,57	79,14	76,78	79,63	79,45	79,61	77,42		78,98	79,33	78,99	77,75	78,92	79,58	79,52	77,40	77,43	79,42	79,42	77,57	96,23	79,49	77,19	77,54	79,27	79,00
14) *Streptomyces xinghaiensis* S187	79,14	79,08	77,95	78,17	76,79	79,08	79,09	77,06	79,05	79,20	79,07	77,44	79,28		79,16	79,30	77,48	79,40	79,08	79,07	77,34	77,98	78,99	79,06	78,02	79,29	79,16	78,10	77,58	95,87	79,53
15) *Streptomyces* sp RTd22	83,35	81,37	76,65	76,64	75,32	83,29	82,62	75,83	83,32	94,77	83,33	76,44	78,35	78,44		78,28	76,86	78,08	83,30	83,48	76,54	76,65	83,58	83,69	76,79	78,27	83,58	76,39	76,83	78,88	78,71
16) *Streptomyces albireticuli* MDJK11	78,76	78,85	77,23	77,37	75,96	78,74	78,84	76,49	78,75	79,04	78,76	77,08	78,93	79,07	79,01		77,44	83,34	78,73	78,80	77,12	77,24	78,67	78,70	77,24	78,82	78,71	77,38	77,29	79,25	84,34
17) *Streptomyces* sp ASO4wet	77,23	77,31	77,00	78,08	76,02	77,19	77,13	76,46	77,19	77,44	77,18	76,54	77,64	77,52	77,30	77,51		77,53	77,15	77,10	76,65	77,07	77,14	77,13	77,05	77,60	77,11	78,29	76,67	77,53	77,54
18) *Streptomyces rectiverticillatus* B15-08	78,86	78,97	77,35	77,53	76,03	78,78	79,07	76,59	78,83	79,09	78,82	77,10	79,08	79,18	78,98	83,49	77,42		78,79	78,86	77,04	77,33	78,80	78,81	77,38	79,21	78,85	77,40	77,20	79,32	83,18
19) *Streptomyces* sp M56	97,84	81,07	76,17	76,25	75,22	98,43	82,69	75,19	98,08	83,00	98,09	76,33	78,16	78,17	82,87	77,61	76,75	77,51		89,83	76,46	76,20	90,38	90,40	76,30	78,06	89,90	76,28	76,58	78,74	78,08
20) *Streptomyces violaceusniger* Tu 4113	90,41	81,70	76,51	76,58	75,12	90,46	83,13	75,44	90,48	83,65	90,49	76,36	78,51	78,33	83,46	78,17	76,87	77,94	90,47		76,54	76,51	94,85	94,88	76,66	78,51	94,46	76,25	76,71	78,73	78,43
21) *Streptomyces* sp XC 2026	77,20	77,13	76,64	77,29	77,76	77,06	77,40	78,13	77,11	77,23	77,11	97,88	77,53	77,23	77,13	77,31	76,66	77,25	77,11	77,16		76,77	77,11	77,13	76,70	77,43	77,08	77,16	86,96	77,32	77,24
22) *Streptomyces* sp CNQ-509	77,29	77,21	94,54	76,87	76,08	77,20	77,20	76,41	77,17	77,31	77,17	76,49	77,64	77,69	77,31	77,53	76,77	77,45	77,20	77,21	76,47		77,21	77,24	97,12	77,52	77,21	77,07	76,75	77,71	77,59
23) *Streptomyces* sp 11-1-2	90,72	81,62	76,48	76,25	75,05	90,85	83,09	75,34	90,88	83,68	90,89	76,25	78,29	78,34	83,55	77,97	76,84	77,69	90,82	94,73	76,42	76,51		98,39	76,44	78,23	94,21	76,19	76,61	78,71	78,31
24) *Streptomyces hygroscopicus* XM201	90,71	81,68	76,43	76,44	75,02	90,84	83,13	75,31	90,80	83,60	90,81	76,25	78,39	78,24	83,57	77,99	76,73	77,88	90,82	94,67	76,52	76,44	98,40		76,48	78,32	94,15	76,25	76,54	78,67	78,36
25) *Streptomyces* sp WAC 06738	77,39	77,27	94,69	76,99	75,90	77,31	77,26	76,38	77,31	77,55	77,29	76,58	77,67	77,69	77,52	77,57	76,83	77,58	77,32	77,39	76,59	97,00	77,18	77,23		77,65	77,33	77,02	76,75	77,65	77,74
26) *Streptomyces buecherae* NA00687	79,09	78,76	77,12	77,48	75,95	79,43	79,03	76,61	79,37	79,23	79,36	77,11	96,12	79,04	79,17	78,83	77,60	78,83	79,40	79,33	77,06	77,19	79,23	79,26	77,39		79,41	76,95	77,30	79,13	78,87
27) *Streptomyces antimycoticus* NBRC 100767	90,57	81,75	76,50	76,75	75,38	90,71	83,35	75,68	90,73	83,90	90,74	76,48	78,74	78,47	83,75	78,23	76,84	78,08	90,69	94,73	76,69	76,60	94,60	94,57	76,73	78,74		76,52	76,74	78,76	78,75
28) *Streptomyces* sp NHF165	77,06	77,07	76,98	80,72	76,13	77,31	77,00	76,71	77,23	77,12	77,23	76,99	77,14	77,87	77,01	77,41	78,22	77,23	77,31	77,07	76,94	76,92	77,05	77,06	76,97	77,09	77,02		76,98	78,01	77,50
29) *Streptomyces harbinensis* NA02264	77,28	77,16	76,75	77,38	77,72	77,21	77,44	78,23	77,26	77,34	77,26	86,95	77,63	77,55	77,25	77,42	76,54	77,27	77,16	77,17	86,92	76,88	77,17	77,17	76,84	77,57	77,16	77,37		77,54	77,29
30) S07_1.15	79,10	79,09	78,05	78,03	76,88	79,07	79,16	77,21	79,00	79,29	79,01	77,56	79,19	**95,83**	79,17	79,45	77,47	79,45	79,09	78,94	77,48	78,07	78,94	79,00	78,03	79,18	79,01	78,08	77,78		79,64
31) *Streptomyces olivoreticuli* ATCC 31159	78,88	78,64	76,99	77,13	75,58	78,86	78,62	75,95	78,84	79,19	78,83	76,87	78,66	78,93	79,08	83,92	77,39	82,46	78,79	78,90	76,89	77,04	78,84	78,93	77,11	78,58	79,02	76,98	77,07	79,36	

Table S5: Presence of putative Marine Adaptation Genes in the sequenced strains.

MAG type	MA3_2.13 gene	MA3_2.13 annotation	S07_1.15 gene	S07_1.15 annotation
ABC transporter	prot_01227	putative acyl-peptide hydrolase		
ABC transporter	prot_05689	Phosphate ABC transporter, permease protein PstA	prot_03574*	Phosphate ABC transporter, permease protein PstC
ABC transporter	prot_03522<br>prot_02557	Phosphate ABC transporter, ATP-binding protein PstB	prot_03572	Phosphate ABC transporter, ATP-binding protein PstB
ABC transporter	prot_03184	Branched-chain amino acid ABC transporter, ATP-binding protein LivG	prot_05342	Branched-chain amino acid ABC transporter, ATP-binding protein LivG
ABC transporter	prot_03185	Branched-chain amino acid ABC transporter, ATP-binding protein LivF	prot_05341	Branched-chain amino acid ABC transporter, ATP-binding protein LivF
ABC transporter	prot_03182	Branched-chain amino acid ABC transporter, permease protein LivH	prot_05344*	Branched-chain amino acid ABC transporter, permease protein LivH
ABC transporter	prot_02414	Hydroxymethylpyrimidine ABC transporter, ATPase component	prot_02525	ABC transporter, ATP-binding protein
ABC transporter	prot_02412	Hydroxymethylpyrimidine ABC transporter, transmembrane component		
Acyl carrier protein	prot_00239<br>prot_06194	3-hydroxybutyryl-CoA dehydrogenase Acyl carrier protein		
ATP-binding protein			prot_02589	hypothetical protein
Channels and pores			prot_05327	hypothetical protein
Chitin-binding protein	prot_06349	Chitin binding protein	prot_04355	Chitin binding protein
DUF3307 domain-containing protein	prot_03023	hypothetical protein		
Electron transport			prot_00069	NADH-ubiquinone/plastoquinone oxidoreductase, chain 3
Electron transport			prot_00067	NADH-ubiquinone oxidoreductase chain H
Electron transport			prot_00066	NAD(P)H-quinone oxidoreductase chain J
Electron transport			prot_00065	NADH-ubiquinone oxidoreductase chain K
Electron transport			prot_00064	NADH-ubiquinone oxidoreductase chain L
Electron transport			prot_00063	NADH-ubiquinone oxidoreductase chain M
Electron transport			prot_00062	NADH-ubiquinone oxidoreductase chain N
Electron transport	prot_04025	Na(+) H(+) antiporter subunit D	prot_06354	Na(+) H(+) antiporter subunit D
Electron transport	prot_04024	Na(+) H(+) antiporter subunit C	prot_06355	Na(+) H(+) antiporter subunit C
Electron transport	prot_04023	Na(+) H(+) antiporter subunit A / Na(+) H(+) antiporter subunit B	prot_06356	Na(+) H(+) antiporter subunit A / Na(+) H(+) antiporter subunit B
Helix-turn-helix domain-containing protein	prot_03940	Transcriptional regulator, IclR family	prot_04216	Transcriptional regulator, IclR family
Helix-turn-helix domain-containing protein	prot_01661	putative DNA-binding protein	prot_03089	Uncharacterized protein with N-terminal Xre-type DNA-binding domain
Helix-turn-helix domain-containing protein	prot_05547	hypothetical protein		
Hydrolase	prot_02379	Fumarylacetoacetase	prot_02773	Fumarylacetoacetase
Hydrolase	prot_00597	Adenosylhomocysteinase		
Hydrolase	prot_02085	amidohydrolase		
Hydrolase	prot_01198	RidA/YER057c/UK114 superfamily protein		
Ion Transport	prot_00622	Sodium/calcium antiporter	prot_02968	Sodium/calcium antiporter
Ion Transport	prot_03982	KtrAB potassium uptake system, peripheral membrane component KtrA	prot_03107	KtrAB potassium uptake system, peripheral membrane component KtrA
Ion Transport	prot_03983*	KtrAB potassium uptake system, integral membrane component KtrB	prot_03108	KtrAB potassium uptake system, integral membrane component KtrB
Isomerase	prot_05444	mandelate racemase family protein Pfl_3283	prot_00114	mandelate racemase family protein Pfl_3283
MFS transporter			prot_00423	Uncharacterized MFS-type transporter
NUDIX domain-containing protein	prot_00596	Adenosylhomocysteinase	prot_02184	NTP pyrophosphohydrolases including oxidative damage repair enzymes
Oxidoreductase	prot_05430	FMN reductase (NADPH)		
Oxidoreductase	prot_06195	3-hydroxybutyryl-CoA dehydrogenase	prot_01237	3-hydroxyacyl-CoA dehydrogenase
Oxidoreductase	prot_00514	Acyl-CoA dehydrogenase	prot_05664	Acyl-CoA dehydrogenase
Oxidoreductase	prot_00878	2,5-dioxovalerate dehydrogenase	prot_00936	2,5-dioxovalerate dehydrogenase
Oxidoreductase	prot_02875	Formaldehyde dehydrogenase MscR, NAD/mycothiol-dependent (EC 1.2.1.66) / S-nitrosomycothiol reductase MscR		
Oxidoreductase	prot_01388	3-oxoacyl-[acyl-carrier protein] reductase	prot_00237	Oxidoreductase, short-chain dehydrogenase/reductase family
Oxidoreductase	prot_04843	Oxidoreductase, short-chain dehydrogenase/reductase family	prot_03132	Oxidoreductase, short-chain dehydrogenase/reductase family
Peptidase	prot_05414	hypothetical protein	prot_00679	hypothetical protein
Phosphatase	prot_00515	FkbH like protein		
Transcriptional regulator	prot_04397	hypothetical protein		
Transferase	prot_01692	Acetyltransferase	prot_00581	Acetyltransferase, GNAT family
Transferase	prot_05262	hypothetical protein		

* Identified using annotation-only (keyword) search

Table S6: Putative biosynthetic gene clusters (BGCs) for isolate MA3_2.13.

#BGC	Localization	Length (kb)	BGC type[a]	Most Similar BGC from MIBiG[b]
1	212807...297030	84,2	hybrid NRPS-T1PKS	
2	555098...599235	44,1	iT1PKS	
3	886399...894213	7,8	other/siderophore	desferrioxamine E - BGC0001478 (50%)
4	939435...949851	10,4	other	ectoine - BGC0000853 (100%)
5	1325419...1343987	18,6	terpene	
6	1464068...1579833	115,8	hybrid NRPS-T1PKS	
7	2787528...2810209	22,7	RiPP/class IV lanthipeptide	
8	3075560...3167749	92,2	NRPS	atratumycin - BGC0001975 (52%)
9	3185683...3228931	43,2	T3PKS	
10	3241764...3263490	21,7	terpene	geosmin - BGC0001181 (100%)
11	3509738...3532272	22,5	RiPP/lasso peptide	
12	3581811...3602363	20,6	terpene	
13	4069820...4096256	26,4	RiPP/class I lanthipeptide	
14	4661162...4702310	41,1	Other	triacsins - BGC0001983 (71%)
15	4969959...5015964	46,0	PKS-like (hglE)	
16	5623436...5637943	14,5	other/siderophore	
17	5754307...5765146	10,8	RiPP	
18	5875209...6035077	159,9	T1PKS	
19	6156127...6204196	48,1	hybrid NRPS-T1PKS	
20	6266254...6302465	36,2	RiPP/thiopeptide	
21	6303108...6391716	88,6	T1PKS	
22	6391965...6521694	129,7	T1PKS	
23	6521785...6625074	103,3	hybrid NRPS-T1PKS	
24	6624965...6705434	80,5	hybrid NRPS-T1PKS	arsono-polyketide - BGC0001283 (79%*
25	6825090...6846932	21,8	terpene	
26	6868736...6909845	41,1	T3PKS	
27	6993789...7031495	37,7	hybrid NRPS-T1PKS	
28	7031391...7054390	23,0	RiPP/class II lanthipeptide	
29	7085908...7195061	109,2	T1PKS	BE-14106 - BGC0000029 (64%)
30	7202972...7252454	49,5	hybrid NRPS-T1PKS	
31	7312007...7422495	110,5	hybrid NRPS-T1PKS	JBIR-100 - BGC0001348 (72%*)
32	7422526...7507697	85,2	hybrid NRPS-T1PKS	neocarzilin A - BGC0000111 (85%*)

a) BGC type as defined in antiSMASH tool

b) Most similar MIBiG BGC (percentage of genes showing similarities to MIBiG BGC). Only BGC sharing gene similarities above 50% are indicated.

* - similarity only with the PKS cluster

Table S7: Putative biosynthetic gene clusters (BGCs) for isolate S07_1.15.

No BGC	localization	Length (kb)	BGC type[a]	Similarity with MIBiG BGC[b]	*Similarity with S. xinghaiensis BGCs* [c]
**1**	55375...78081	22,7	RiPP/lasso peptide	citrulassin B - BGC0001549 (100%)	43% (Las1)
**2**	504455...585511	81,1	NRPS	isocomplestatin - BGC0000326 (93%)	100% (Nrps1)
**3**	684602...702094	17,5	terpene		93% (Tep1)
**4**	745877...754214	8,3	RiPP		16% (Bac1)
**5**	950051...983589	33,5	other/saccharide	neomycin - BGC0000709 (50%)	96% (Neo1)
**6**	1031310...1056553	25,2	RiPP/class i lanthipeptide		26% (Lan1)
**7**	1068441...1093528	25,1	terpene	hopene - BGC0000663 (53%)	95% (Tep2)
**8**	1169531...1185264	15,7	terpene		90% (Tep3)
**9**	1265412...1301282	35,9	RiPP/thiopeptide		100% (Thio1)
**10**	1413432...1421436	8,0	Other/siderophore	desferrioxamine E - BGC0001478 (100%)	71% (Sid1)
**11**	1499427...1508922	9,5	RiPP		100% (Bac2)
**12**	1667469...1709648	42,2	RiPP/class v lanthipeptide		-
**13**	1775028...1787157	12,1	Other/siderophore		100% (Sid4)
**14**	2185930...2206765	20,8	RiPP/class iii lanthipeptide		100% (Lan2)
**15**	3247887...3271645	23,8	terpene	isorenieratene - BGC0001456 (87%)	85% (Tep4)
**16**	3905019...3945332	40,3	other/phenazine		-
**17**	4701256...4776588	75,3	T2PKS		76% (Pks2)
**18**	4995441...5006276	10,8	RiPP		-
**19**	5490378...5513110	22,7	RiPP/class iii lanthipeptide	SapB - BGC0000551 (100%)	LanKC
**20**	5855823...5867586	11,8	other/siderophore		100% (Sid3)
**21**	6009580...6019984	10,4	Other	ectoine - BGC0000853 (100%)	100% (Ect1)
**22**	6637363...6659228	21,9	terpene		-
**23**	7006369...7047466	41,1	T3PKS		89% (Pks3)
**24**	7052668...7064578	11,9	RiPP		90% (Bac3)

a) BGC type as defined in antiSMASH tool

b) Most similar MIBiG BGC (percentage of genes showing similarities to MIBiG BGC). Only BGC sharing gene similarities above 50% are indicated.

c) BGC defined in [24]

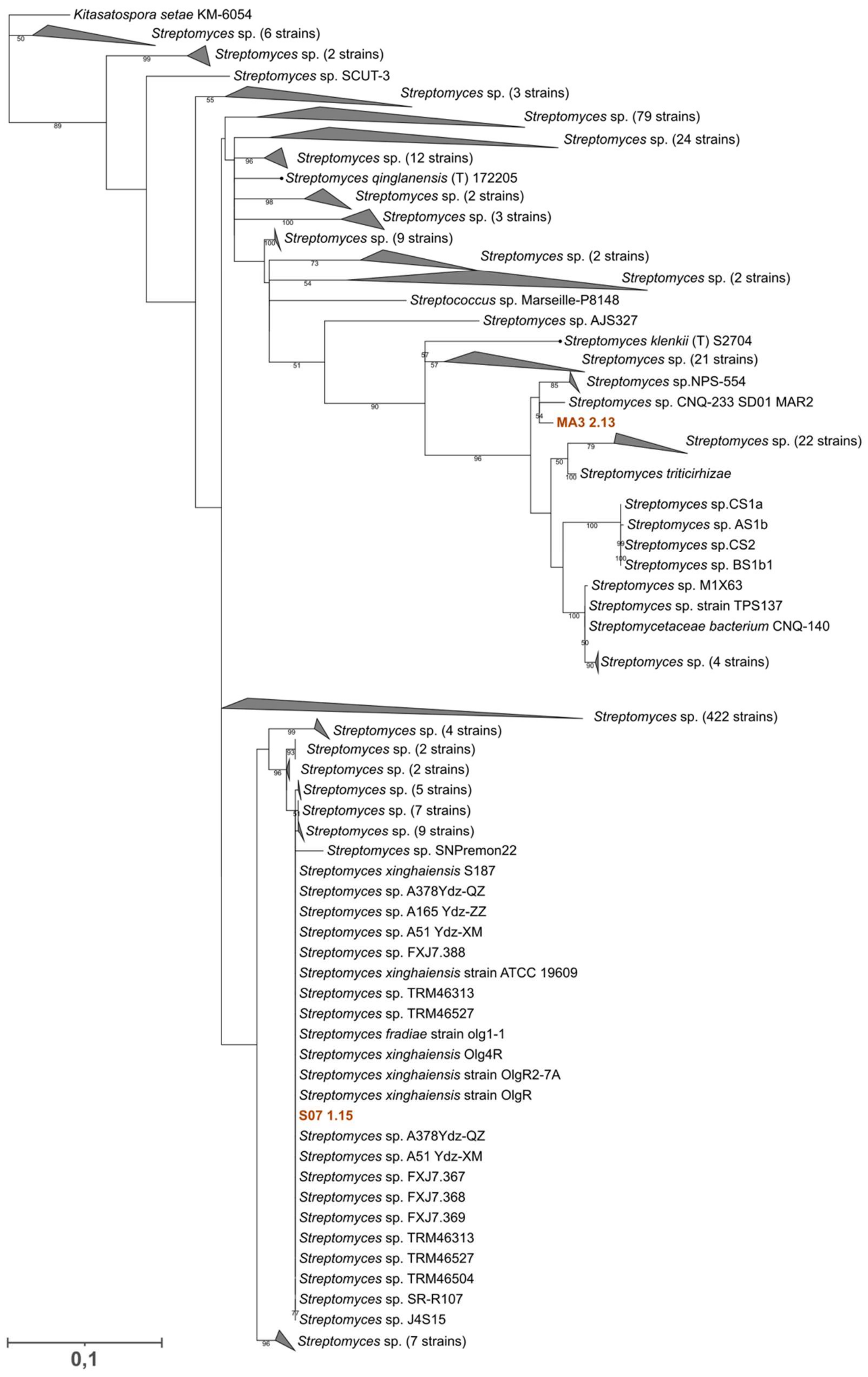

Figure S1: Maximum likelihood phylogenetic tree (GTR+G+I) of the 16S rRNA sequences of isolates MA3_2.13 and S07_1.15 (highlighted in red) and 683 *Streptomyces* sp. *Kitasatospora setae* KM-6054 was used as outgroup. Bootstrap values from 1000 replications are shown at branch points. Bar, 0.1 substitution per site.

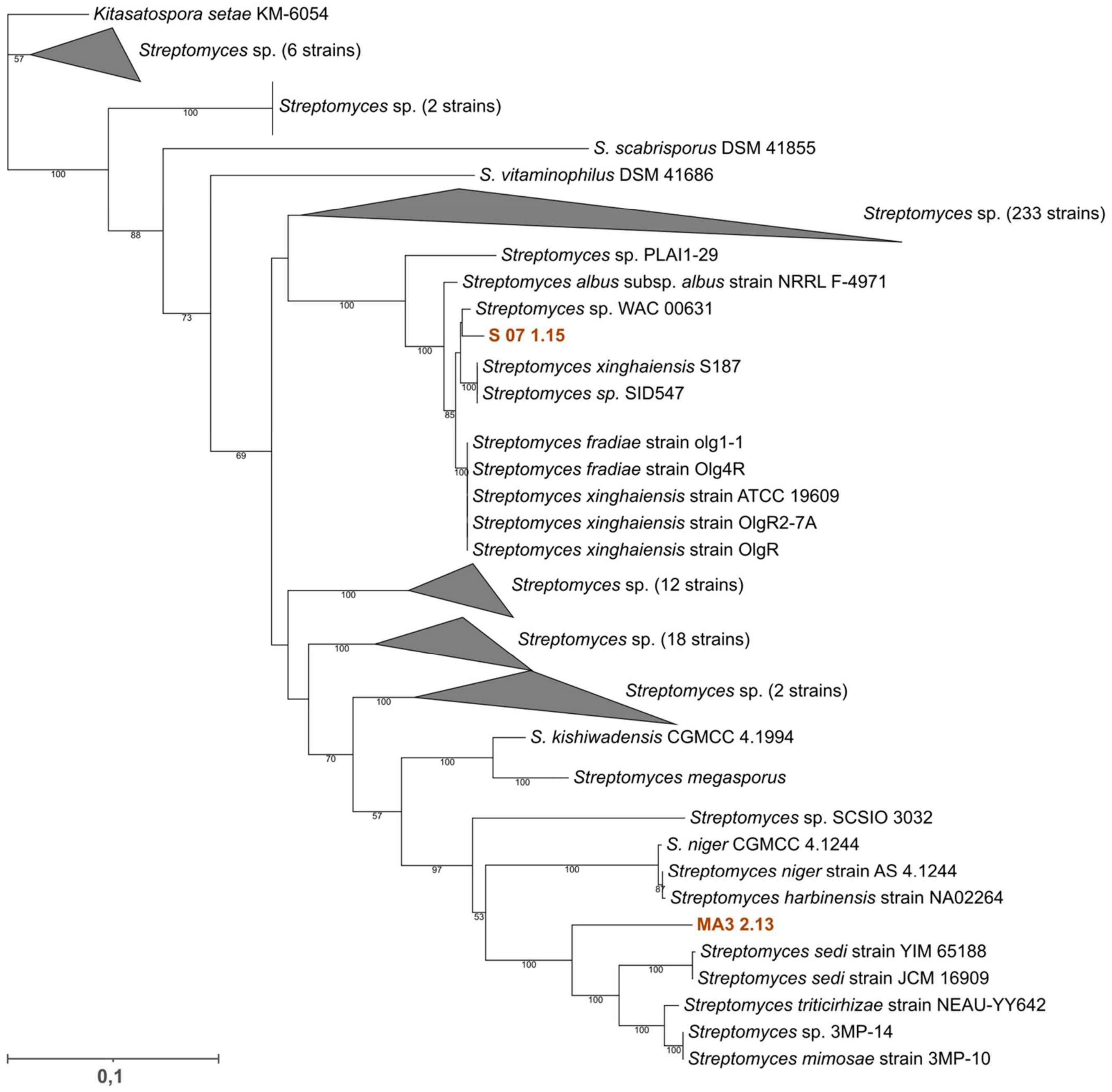

Figure S2: Maximum likelihood phylogenetic tree (GTR+G+I, 2000 Bootstrap replicates), based on five gene sequences (*atpD*, *gyrB*, *recA*, *rpoB* and *trpB*) concatenated manually, showing the phylogenetic relationship of isolates MA3_2.13 and S07_1.15 with members of the *Streptomyces* genus (N=300). *Kitasatospora setae* KM-6054 was used as outgroup. Portions of the tree collapsed are labelled and numbers represent the number of strains in the collapsed subtrees.
